# Evaluation of 3D Printing of Cereal–Legume Starch-Based Gels Formulated with Red Adzuki Bean and Germinated Brown Rice Flour

**DOI:** 10.3390/foods14101791

**Published:** 2025-05-18

**Authors:** Ran Liu, Yu Zhuang, Jiakai Song, Liuyang Shen, Yanling Yin

**Affiliations:** 1 College of Engineering, Northeast Agricultural University, Harbin 150030, China; yagyuu@foxmail.com (R.L.); zy193123@neau.edu.cn (Y.Z.); songjk040803@163.com (J.S.); 2 College of Life Sciences, Tarim University, Alar 843300, China; 3 College of Life Sciences, Northwest A & F University, Xianyang 712100, China

**Keywords:** 3D food printing, germinated brown rice, red adzuki bean, starch-based gel

## Abstract

Three-dimensional (3D) food printing (3DFP) is an emerging technology that enables the creation of personalized and functional foods by precisely controlling nutritional content and shape. This study investigated the 3D printability and rheological behavior of cereal–legume starch-based gels formulated with germinated brown rice (GBR) and red adzuki bean (RAB) flours, supplemented with xanthan and guar gums as functional additives. The physicochemical and structural properties of the gels were characterized through FT-IR, rheology, texture analysis, SEM, and sensory evaluation. In addition, the 3D printing fidelity, rheological behavior, color attributes, textural properties, microstructure, and sensory scoring of the printed products were evaluated. The results indicated that the gels exhibited pseudoplastic behavior, with the RABF/GBRF ratio of 1:2 (RG1:2) formulation showing optimal color properties (ΔE* = 0.60 ± 0.86) and the RABF/GBRF ratio of 2:1 (RG2:1) formulation demonstrating superior printing fidelity and structural stability (printing accuracy = 99.37 ± 0.39%). The gels’ mechanical properties, such as hardness and chewiness, were significantly influenced by the RABF and GBRF ratios, with RG2:1 exhibiting the highest hardness (1066.74 ± 102.09) and RG1:2 showing the best springiness (0.64 ± 0.10). The sensory evaluation results indicated that the RABF/GBRF ratios of 1:1 (RG1:1) and RG1:2 had relatively high overall acceptance scores. These findings indicate that specific ratios of RABF and GBRF improve the 3D printability and textural properties of cereal–legume starch-based gels, enhancing their suitability for 3D food printing applications. This study provides valuable insights into the development of personalized and functional cereal–legume starch-based foods using 3DFP technology.

## 1. Introduction

Three-dimensional (3D) food printing, also known as food layering manufacturing technology, is a digitally controlled process that uses 3D solid models as a basis and enables food materials to be printed layer by layer to create complex and varied food products by controlling temperature or pressure [1,2,3]. Three-dimensional food printing (3DFP) technology allows the customization of food products to meet individual physiological and nutritional needs by modifying nutrient content, reducing undesirable compounds, and incorporating health-promoting components such as fiber, protein, and phytochemicals [4,5]. Therefore, 3DFP technology can not only enrich food styles and improve food quality but also precisely regulate the nutritional content of food to meet the health needs and taste preferences of different individuals (such as the elderly, children, and athletes) [6,7]. Meanwhile, the use of 3D printing technology enables the realization of complex shapes and structures that are difficult to construct by traditional processing methods [8]. Compared to traditional food production, 3DFP technology addresses the growing demand for personalized and functional foods and has great development prospects in the food processing and manufacturing industry [7].

Cereals and legumes are essential components of the human diet and play a pivotal role in global dietary patterns [9]. Germinated brown rice (GBR), a representative cereal grain, and red adzuki beans (*Vigna angularis*) (RAB), a representative legume, are valuable resources for food development due to their rich starch content and diverse bioactive compounds [10,11]. Three-dimensional food printing technology offers significant advantages in achieving personalized product design, precise nutritional formulation, and accurate dosage control, making it particularly suitable for the innovation of cereal–legume starch-based food products [12]. Currently, a wide range of materials, including chocolate [13], cheese [14], cereals [15], and ground meat [16], have been successfully applied in food 3D printing. Through straightforward modifications and nutritional optimization of these materials, it is feasible to produce healthier 3DFP products [17]. For example, Khemacheevakul et al. [18] employed dual-extrusion 3D printing technology to fabricate chocolate layers with varying sugar concentrations, effectively reducing overall sugar content while maintaining optimal sweetness perception, thereby creating a healthier low-sugar chocolate alternative. Similarly, Riantiningtyas et al. [19] utilized 3DFP technology to develop protein-enriched desserts using yogurt–gel inks with different concentrations of gelatin and whey protein isolate (WPI). Their findings demonstrated that increased gelatin content enhanced gel strength, while WPI contributed to gel softening but improved post-printing shape stability, with optimal gelatin-WPI combinations achieving balanced texture, appearance, and sensory properties. Furthermore, Yu et al. [20] conducted a comprehensive investigation of the effects of varying konjac gum to xanthan gum ratios on the rheological properties, printability characteristics, textural attributes, microstructure formation, and protein–glycan interactions of cooked sturgeon paste. This research ultimately led to the development of a textured sturgeon paste suitable for individuals with swallowing difficulties. Despite these advancements, research on the 3DFP of GBR and RAB flour remains limited. Given their high content of starch, dietary fiber, protein, and other nutritional components, coupled with the excellent gelation and gelatinization properties of starch-based foods, GBR and RAB present promising potential as 3DFP materials [21].

The extrusion-based 3DFP technique is one of the common food 3D printing technologies [22]. In food 3D printing, the selection of raw materials plays a pivotal role in determining the quality characteristics of printed products [23]. To enhance printability, base materials, particularly cereal and legume flours, are typically combined with food hydrocolloids such as xanthan gum and guar gum to formulate composite gels with optimal 3D printing properties [20]. Among various food hydrocolloids, xanthan gum exhibits distinctive rheological properties, demonstrating excellent water solubility and remarkable stability under diverse conditions, including heat, acid, and alkaline environments. These characteristics enable its versatile application as a thickener, emulsifier, and stabilizer in the food industry [24]. Similarly, guar gum, a high-molecular-weight natural hydrocolloid, serves as one of the most prevalent quality enhancers and natural thickeners in food processing [25]. The incorporation of appropriate quantities of xanthan and guar gums not only optimizes nutritional profiles and enhances textural properties but also significantly improves the structural stability of 3DFP products [26].

This study adopts an attempt by maintaining consistent levels of food additives while systematically modifying the proportions of primary food components to facilitate 3D food printing. Specifically, GBR and RAB flours were selected as base materials, supplemented with xanthan gum and guar gum, to develop a cereal–legume starch-based food gel designed for extrusion-based 3DFP. The specific objectives of the current study are as follows: (1) to investigate the gelation characteristics of GBR and RAB flour-based gels with varying formulation ratios and (2) to assess the 3D printing performance and resultant product quality of GBR and RAB flour-based gel foods.

## 2. Materials and Methods

### 2.1. Preparation of the Gel

The preparation of GBR was conducted with reference to Zhu et al. [27], and the RAB samples were supplied directly by the Processing Research Institute, Heilongjiang Academy of Agricultural Sciences. The freeze-dried germinated brown rice flour (GBRF) and red adzuki bean flour (RABF) were sifted using an 80-mesh sieve.

**Figure 1:**
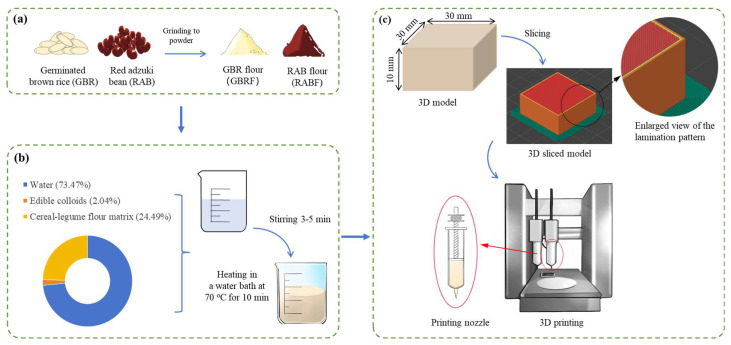
Flowchart of gel preparation and 3D printing process. (**a**) Preparation of GBR flour and RAB flour; (**b**) preparation of cereal–legume starch-based gel; (**c**) 3D printing process of the gel.

The preparation of the cereal–legume starch-based gel was adapted from Huang et al. [28] with appropriate modifications and refined based on preliminary experiments. The flowchart of the gel preparation and 3D printing process is illustrated in Figure 1.

The RABF and GBRF in different proportions (Table 1) were used as base materials. These were subsequently combined with a hydrocolloid mixture consisting of 2% xanthan gum (XG) and guar gum (GG), maintaining a xanthan gum to guar gum (XG-GG) ratio of 2:5 (*w*/*w*). The blend was then hydrated with boiling distilled water (98.0 ± 0.3 °C) at a material-to-water ratio of 1:3 (*w*/*w*), followed by thorough mixing for 3–5 min to ensure homogeneous distribution. To enhance molecular interactions and facilitate network formation, the mixture underwent thermal stabilization in a 70 °C water bath for 10 min. Following thermal treatment, the samples were cooled to ambient temperature, resulting in the formation of gel samples, which were subsequently stored at 4 °C for further experimental analysis. All experiments were carried out at room temperature (25 ± 1 °C).

An experimental scheme shown in Table 1 was developed based on the existing literature reported by Yu et al. [20] and our preliminary experimental results. Based on these trials, three representative ratios of RABF/GBRF—1:1, 1:2, and 2:1—were selected for gel preparation and subsequent analysis.

### 2.2. Characterization of Gel Properties

In order to evaluate the characteristics of the prepared gel, the chemical interactions between materials and XG-GG, rheological properties, and the microstructure of the gel were characterized and analyzed.

#### 2.2.1. Fourier Transform Infrared Spectroscopy (FT-IR)

The FT-IR spectra of the gel were measured using an FT-IR spectrometer (IRAffinity-1S, Shimadzu, Kyoto, Japan) according to the method described by Zhu et al. [27]. The spectrum was recorded over a wavenumber range of 400 to 4000 cm^−1^ with a resolution of 4 cm^−1^. A KBr blank tablet was used as a reference.

#### 2.2.2. Static and Dynamic Rheological Properties

A rotational rheometer (Discovery HR-2, TA Instruments, Inc., New Castle, DE, USA), equipped with 20 mm parallel plates and 1000 μm spacing, was used to perform full rheological characterization of the gel. The experimental operation was adapted from the previous methods with necessary modifications [28]. Water evaporation was prevented by sealing the edges with silicone oil and preheating at 25 °C for 5 min to reach equilibrium. Static rheology was scanned at shear rates from 0.1–10 s^−1^ to resolve hydrodynamic properties. For dynamic rheological analysis, frequency sweep tests were conducted within the range of 0.1–10 Hz at a constant strain of 2%. Creep tests were performed at 30 Pa for 300 s to characterize the material stress response. The obtained shear curves were fitted using Equation (1) [29]:

(1) $$\eta =K\gamma^{n-1}$$

where *η* is the viscosity of the gel sample (Pa·s); *γ* is the shear rate (s^−1^); K is the consistency (Pa·s); and *n* is the power-law index. For a pseudoplastic solution, *n* < 1.

Similar to the static rheological measurements, in dynamic rheological measurements, the storage modulus (*G*′) versus loss modulus (*G*″) and the tangent value of the loss angle (tanδ) were obtained as a function of frequency by scanning at 1–100 rad/s with 0.1% strain [30] and were used to assess elastic and viscous properties within the linear viscoelastic region. The frequency function tanδ is defined by Equation (2) [30]:

(2) $$\tan\delta =\frac{G^{\prime \prime }}{G^{\prime }}$$

#### 2.2.3. Scanning Electron Microscopy (SEM)

SEM (S-3400 N, Hitachi, Tokyo, Japan) was used to observe the microstructural morphology of the gel according to Shen et al. [31]. Briefly, the sample was cut into ultra-thin slices and then uniformly adhered to conductive tape, followed by the application of a gold coating to their surfaces. Micrographs of the gel were then taken at an accelerating voltage of 5 kV, and the microstructural changes were observed at a magnification of 1500×.

### 2.3. Color Measurement of the Gel

The color of the gel was measured by using a colorimeter (CR-20, Konica Minolta Inc., Tokyo, Japan). The color scale was expressed in terms of L*, a*, and b* based on the CIE-Lab color space [31]. L* represents brightness or darkness between 0 (black) and 100 (white), and a* represents redness (+) or greenness (−), while b* represents yellowness (+) and blueness (−). Based on these, the total color difference (ΔE*) can be further calculated:

(3) $$\Delta E=\sqrt{{\left(L_{0}^{*}-L^{*}\right)}^{2}+{\left(a_{0}^{*}-a^{*}\right)}^{2}+{\left(b_{0}^{*}-b^{*}\right)}^{2}}$$

where *L*_0_*, *a*_0_* and *b*_0_* are the color values of the initial samples; L*, a*, and b* are the color values of treated samples.

### 2.4. 3D Printing Process of the Gel

The 3D printing performance of the gel was assessed using an extrusion-based food 3D printer (FoodBot-D1, Shiyin Technology Co., Ltd., Hangzhou, China). A rectangular model measuring 30 mm in length, 30 mm in width, and 15 mm in height, designed in SolidWorks software (Ver. 2024, Dassault Systemes, Waltham, MA, USA), served as the printing model. Prior to printing, the printer parameters were configured via Slic3r software (Ver. 1.2.9) with the following settings: nozzle diameter of 1.2 mm, initial layer height of 0.84 mm, printing speed of 25 mm/s, concentric fill pattern, and 90% fill density.

#### 2.4.1. Determination of Printing Fidelity

The printed samples were stored in an airtight glass container at 4 °C for 0, 0.5, 1, 1.5, and 2 h, after which their dimensions (length, width, and height) were measured. Shape stability was evaluated using the shape stability index (SSI), as defined in Equation (4) [32]

(4) $$SSI(\%)=\frac{Sample\;size\;before\;treatment}{Sample\;size\;after\;treatment}\times 100$$

where sample size before treatment represents the volume before deformation; sample size after treatment refers to the volume after deformation.

#### 2.4.2. Determination of Gel Texture

To comprehensively evaluate the textural properties of the 3D-printed samples, a texture analyzer (Stable Micro System Ltd., TA.XT Plus, Surrey, UK) was employed to conduct a two-cycle texture profile analysis [33]. A cylindrical probe (P/50, 50 mm diameter) was used to compress the samples to 45% of their original height, ensuring adequate assessment of material deformation. The trigger force was set to 5 g for precise and gentle probing. During the test process, a pre-test speed of 1 mm/s, test speed of 5 mm/s, and post-test speed of 5 mm/s were set to ensure consistent and reliable data collection. The textural parameters, including hardness, adhesiveness, springiness, cohesiveness, stickiness, and chewiness, were recorded according to TPA curve attributes [34].

### 2.5. Sensory Evaluation

The experimental methods outlined by Li et al. [35] and Islam et al. [36] were referenced and appropriately modified for sensory evaluation. A total of ten trained participants, five females and five males, were selected for the sensory evaluation, which was repeated three times. Each participant was randomly assigned to samples of different formulations. Sensory evaluation indicators included color, smell, taste, texture, appearance, and overall acceptability, as shown in Table 2. The rating scale ranges from 0 to 8, where “0” indicates that the sample is unacceptable and “8” indicates that it is highly liked. The final score was the average of the results collected from all participants.

### 2.6. Statistical Analysis

The test results were expressed as the mean ± standard deviation (SD) based on three replicates. The OriginPro software (Ver. 2023b, OriginLab Corp., Northampton, MA, USA) was introduced for the graphical representation of the data, and SPSS statistical software (Ver. 19.0, SPSS Inc., Chicago, IL, USA) was used for statistical analysis.

## 3. Results and Discussion

### 3.1. FT-IR Analysis of the Gel

**Figure 2:**
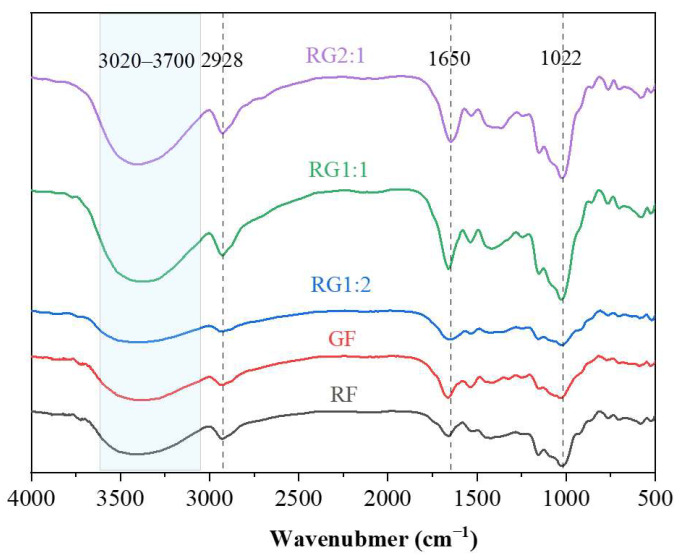
FT-IR spectra of the gel prepared by various formulations. Note: The meanings of the symbols for gel types are detailed in Table 1.

FT-IR spectroscopy has been extensively utilized in studying organic functional groups, as it enables the identification and determination of specific functional groups through their corresponding unique absorption peaks [37]. The FT-IR spectra of the gel prepared by various formulations are shown in Figure 2.

The five various samples exhibited similar characteristic peaks, indicating that despite the differences between GF, RF, and their mixtures, they retained the typical chemical bonding modes within the gel system. The combination of GF and RF did not alter their functional groups or chemical bonding characteristics [27]. The broad peak at 3020–3700 cm^−1^ corresponds to the stretching vibrations of hydroxyl groups in starch molecules, indicating hydrogen bond strength in the samples. Compared to GF and RF, the intensity of this broad peak in RG samples was significantly enhanced. This suggested that combining the two flours (RABF and GBRF) formed stronger hydrogen bonds within the gel system than using either flour alone [38]. As a result, the gel structure remained more intact during the printing process.

Interestingly, as the proportion of GBRF increased (RG1:2 group), the intensity of the tensile vibration peak (3020–3700 cm^−1^) corresponding to hydroxyl groups decreased. This was attributed to the complex interactions between GBRF starch and the gel system. The peak at 1650 cm^−1^ was associated with the C-O stretching vibrations of the amide group in proteins. The peak at 2928 cm^−1^, related to -OH and -CH hydrogen bonds indicated the formation of starch–lipid complexes [39]. Additionally, the peak at 1022 cm^−1^, attributed to the stretching and bending vibrations of CO-, C-C, and OH- groups, as well as the asymmetric stretching of C-O-C glycosidic bonds, suggested interactions between lipids and proteins [40]. Based on the FT-IR spectrum, it was evident that no functional groups disappeared, and no new ones appeared in 3DFP, indicating that no new substances were generated during the process [41]. This further suggested that during the 3D printing process, only changes in the crystal structure took place, as opposed to alterations in the chemical structure, thereby guaranteeing the safety of the 3D-printed food products.

### 3.2. Static Rheological Properties

**Figure 3:**
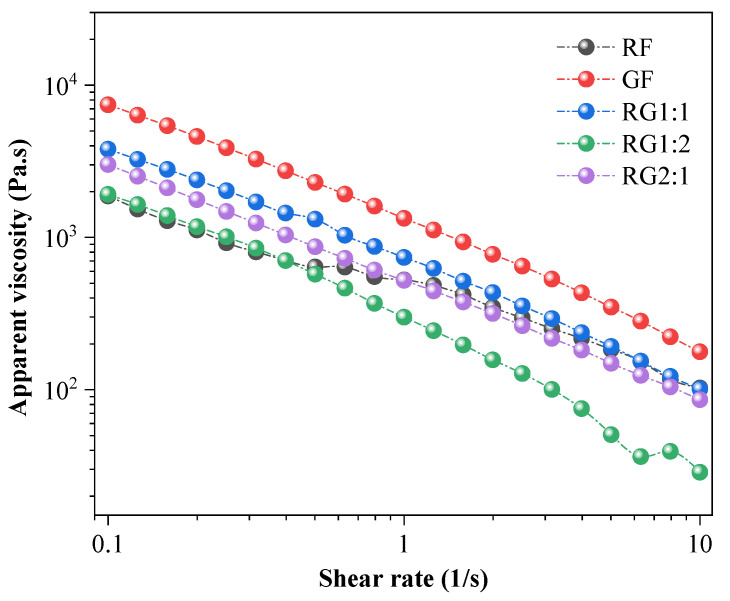
Apparent viscosity versus shear rate profile for gels at different formulations. Note: The meanings of the symbols for gel types are detailed in Table 1.

To understand the viscoelastic properties of gels used as 3D printing materials, Figure 3 illustrates how the apparent viscosity of gels with different formulations changes with shear rate, examining their steady shear rheological characteristics.

From Figure 3, the apparent viscosity, as a key characteristic of pseudoplastic behavior, of the gels with different amounts of added GBRF and RABF decreased with the increase in shear rate. This indicated that the prepared gels showed pseudoplastic fluid properties and exhibited shear thinning behavior [42]. This may be due to the mutual entanglement between starch molecular chains; when the shear force increased, the structure between starch molecular chains underwent significant changes due to the change in the orientation of the large molecular chains, resulting in a decrease in flow resistance, that is, a decrease in apparent viscosity [43]. The quantitative confirmation was further supported by the fitted parameters in Table 3. Specifically, all samples exhibited flow behavior index (*n*) values within the range of *n* < 1, which was a widely recognized indicator of pseudoplastic (shear-thinning) fluids [44]. Moreover, the apparent viscosity for the GF gel reached the highest value, and the strength of the gel system was the highest. This was due to the formation of a denser network structure, which in turn increased the apparent viscosity [42]. The denser network structure in the GF samples could be observed through the SEM images in Section 3.4. The denser structure was attributed to specific interactions among the components in the GF formulation, potentially promoting tighter molecular entanglements and reducing free water mobility, where the similar findings were also reported by Liu et al. [45]. According to Equation (1), the relationship between apparent viscosity (*η*) and shear rate (γ) for the gels with different formulations was fitted to obtain the non-Newtonian exponent *n* and consistency coefficient *K*, as shown in Table 3.

From Table 3, nearly all correlation coefficients (*R*^2^) were higher than 0.98, showing a strong power-law dependence of viscosity on shear rate [29]. In 3D printing processes, fluids begin to exhibit shear-thinning behavior when the power-law index *n* falls below 1 (*n* < 1). Mild shear-thinning characteristics were observed as *n* approaches 0.6, while significant shear-thinning effects became prominent when the index reached values of *n* ≤ 0.2 [46,47]. This suggested that to some extent, the smaller the value of *n*, the stronger the pseudoplasticity.

As shown in Table 3, the *n* values of all gel samples were between 0.2 and 0.3, demonstrating that both RABF and GBRF as well as their composite gels exhibited superior pseudoplasticity. Notably, the RG1:1 and RG1:2 formulations displayed comparable and lower *n* values, suggesting that these materials maintained higher viscosity under low shear conditions while demonstrating reduced viscosity and enhanced flow properties at elevated shear rates [48]. This rheological behavior was particularly advantageous for 3D printing applications, as it aligned well with the dynamic force variations experienced by the feedstock during the printing process, thereby facilitating material extrusion [49]. The parameter *K* indicates the material’s consistency. A lower *K* value facilitates smoother material flow during the 3D printing process, thereby enhancing printing accuracy [50].

### 3.3. Dynamic Rheological Properties

**Figure 4:**
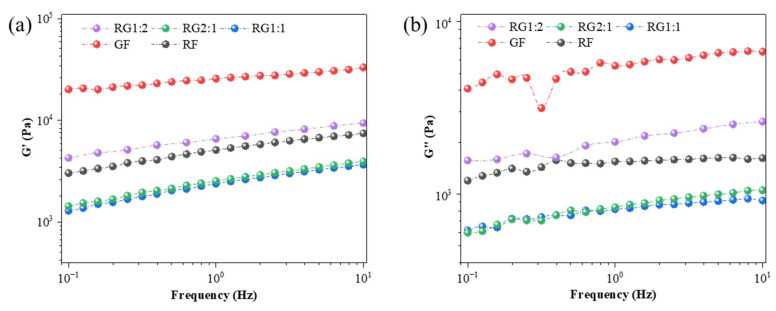
Changes in dynamic rheological properties of the gels with different formulations. (**a**) Storage modulus of the gel (*G*′). (**b**) Loss modulus of the gel (*G*″). Note: The meanings of the symbols for gel types are detailed in Table 1.

Figure 4 presents the dynamic rheological curves of the gels with different formulations, and the storage modulus (*G*′) and loss modulus (*G*″) employed to characterize the viscoelastic properties of the materials [51]. In general, both *G*′ and *G*″ increased with the increase in frequency, and *G*′ was found to be higher than *G*″, meaning a solid-like behavior of materials, reflecting the mechanical strength [30] and indicating that all samples were self-supporting and formed weak gels [51]. *G*′ represents the behavior of an elastic solid and reflects the mechanical strength of the material. Materials with higher *G*′ values typically exhibited superior mechanical strength and were more capable of preserving the printed geometry over time [30]. Among the tested samples, the *G*′ followed the order: GF > RG1:2 > RF > RG2:1 > RG1:1, aligning well with the apparent viscosity results discussed previously. A higher *G*′ value denoted improved mechanical robustness and structural resilience under external stress, which was particularly critical for ensuring dimensional fidelity in 3D-printed constructs.

A lower loss modulus *G*″ indicated reduced energy dissipation as heat during deformation, and heat loss was reduced. In this dimension, the ranking of GF > RG1:2 > RF > RG2:1 > RG1:1 also held true, with the RG1:1 gel performing particularly well in this respect, showing the advantage of energy conversion efficiency during the deformation process [52]. In addition, we calculated tanδ values (i.e., *G*″/*G*′). When the tanδ value approached zero, the properties of the sample were close to those of an ideal elastic solid. Conversely, when the tanδ value approached positive infinity, the properties of the sample were close to those of an ideal viscous fluid [53]. The calculated tanδ value was significantly lower than 1 and ranged from 0.2 to 0.5, confirming that all samples exhibited significant elasticity properties, albeit with some fluctuations. In particular, RG1:2, which indicated that this gel had the highest solid-like properties and the best moldability [54].

### 3.4. Microstructure Changes of the Gel

**Figure 5:**
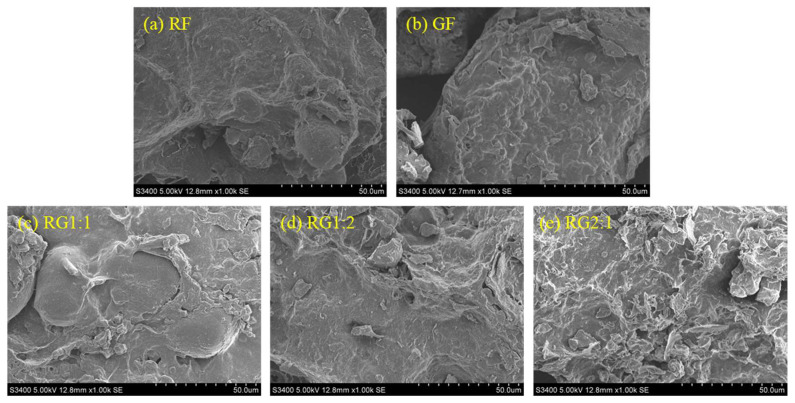
Microstructural changes in 3D-printed samples with different formulations at 1000× magnification. (**a**) RF, RABF; (**b**) GF, GBRF; (**c**) RG1:1, RABF to GBRF ratio of 1:1; (**d**) RG1:2, RABF to GBRF ratio of 1:2; (**e**) RG2:1, RABF to GBRF ratio of 2:1. Note: The meanings of the symbols for gel types are detailed in Table 1.

Figure 5 presents the microstructural morphology of gels with varying formulations at 1000× magnification, revealing significant differences in their structural characteristics. As depicted in Figure 5a,b, both RF and GF exhibited a dense architecture characterized by a textured surface and minimal porosity, suggesting a homogeneous distribution of starch granules within raw RAPF and GBRF matrices. In contrast, Figure 5c,d demonstrate that RG1:1 and RG1:2 possessed a more heterogeneous and disorganized morphology, albeit with a smoother surface texture. These samples displayed noticeable fractures and insufficient interparticle connectivity [55]. Notably, RG2:1 presented a less consolidated structure featuring a coarse surface and prominent pore formation, indicative of an emerging dense structural framework. These observations underscored the substantial influence of starch content in RAPF and GBRF on the microstructural properties of 3D-printed gel specimens. This phenomenon could be attributed to the enhanced molecular interactions between water and starch components at higher starch concentrations, which consequently reinforced the gel network and facilitated the development of a more compact structural configuration [32].

### 3.5. Color Changes in the Gel

The visual color of the final product serves as one of the important reference criteria for consumers when selecting the optimal gel formulation. Table 4 shows the color changes in the gels and the actual color of the 3D-printed samples.

In terms of colorimetric properties, the GBRF-based gel (GF group) exhibited the highest brightness (L* value), while the RABF-based gel (RF group) showed the lowest brightness. Interestingly, when RABF and GBRF were mixed at a ratio of 1:2 (RG1:2 group), the resulting cereal–legume starch-based gel achieved the highest L* value, indicating optimal brightness. Regarding the a* value, which represents the red-green spectrum, the RF group had the highest value, suggesting a stronger red component, whereas the GF group had the lowest. The RG1:2 group demonstrated the lowest a* value, indicating a more balanced color profile. Similarly, the b* value, representing the yellow–blue spectrum, was highest in the GF group and lowest in the RF group, with the RG1:2 group showing a relatively low b* value. The color difference (ΔE*) was highest in the RG2:1 group and lowest in the RG1:1 group, with the RG1:2 group exhibiting a relatively low ΔE* value. Overall, the RG1:2 group demonstrated superior color properties, as evidenced by its balanced L*, a*, and b* values, as well as a low ΔE*.

The observed color differences can be attributed to the varying pigment compositions and interactions between the RABF and GBRF components. GBRF, rich in chlorophyll and carotenoids, likely contributes to higher brightness (L*) and yellowness (b*), while RABF, containing anthocyanins, may enhance the red component (a*). The optimal color properties of the RG1:2 group suggested a synergistic effect where the pigments from both sources interact to produce a visually appealing color. This phenomenon aligned with previous findings [56], where the blending of different plant-based materials often resulted in improved color stability and aesthetic appeal due to the complementary nature of their pigments [57]. Further research into the specific pigment interactions and their stability under different processing conditions could provide deeper insights into the mechanisms behind these colorimetric outcomes.

### 3.6. 3D Printing of the Gel

#### 3.6.1. Printing Fidelity

In order to further evaluate the printing accuracy of the gel, the length, width, and height of the 3D-printed gel were measured, and then the shape stability index (SSI) value was calculated. The closer the SSI value is to 100%, the higher the accuracy, and the higher the printing fidelity [58]. Table 5 shows the changes in SSI values of the 3D-printed gel with different standing times from 0.5 to 2 h.

As demonstrated in Table 5, the SSI of cereal–legume starch-based gels exhibited significant variations depending on the formulation and standing time. The GBR-based gel (GF group) displayed the highest SSI value, while the RAB-based gel (RF group) showed the lowest SSI value across standing times ranging from 0.5 to 2 h. Notably, when RABF and GBRF were mixed at a ratio of 2:1 (RG2:1 group), the resulting gel achieved the highest printing fidelity. Although the sample volume gradually increased over time compared to the initial volume at 0 h, likely due to reduced internal filling density and diminished line support, the SSI value remained within a narrow range of 2% even after 2 h of placement. This suggested that the RG2:1 formulation exhibited excellent structural stability and minimal syneresis, making it particularly suitable for applications requiring short-term storage. The superior stability of the RG2:1 gel could be attributed to the synergistic interaction between RABF and GBRF, which may increase the mobility of tightly and weakly bound water and decreases the mobility of free water in fresh starch gel systems, a phenomenon consistent with the behavior of biopolymer-based gels under static conditions [59,60]. These findings indicated that the RG2:1 formulation was well-suited for producing skeletonized structures with enhanced stability and adaptability to short-term storage, offering promising potential for applications in 3D food printing and functional food design.

#### 3.6.2. Texture Properties

**Figure 6:**
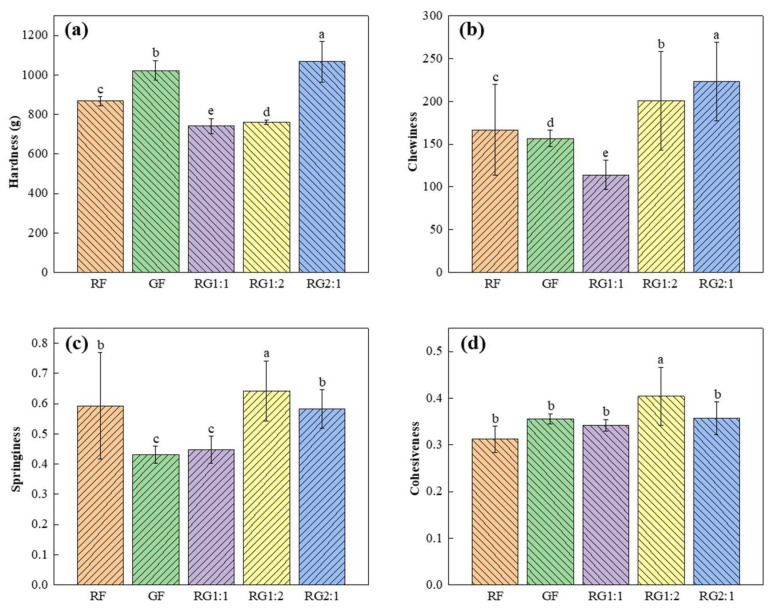
Changes in textural characteristics of 3D-printed gel samples. (**a**) Hardness; (**b**) chewiness; (**c**) springiness; (**d**) cohesiveness. Different letters in the same column indicate significant differences between samples (*p* < 0.05). Note: The meanings of the symbols for gel types are detailed in Table 1.

The textural properties of food products, particularly hardness, chewiness, springiness, and cohesiveness [61], serve as essential determinants of consumer acceptance. As quantified in Figure 6, these parameters demonstrated significant variations across samples, suggesting distinct structural and compositional differences in food matrices.

Hardness, defined as the peak force required to achieve a specified deformation, was a critical parameter reflecting the supportability of gel structures [62] and exhibited statistically significant differences (*p* < 0.05) between formulations (Figure 6a). Sample RG2:1 demonstrated maximum hardness (1066.74 g), while RG1:1 showed minimum values (742.45 g). This variation likely stemmed from differential molecular interactions in the protein–polysaccharide matrix, where higher RG2:1 hardness indicated enhanced cross-linking between biopolymer chains through hydrogen bonding or hydrophobic interactions [63]. The inverse relationship between water activity and hardness in RG1:1 may reflect plasticizing effects of free water molecules disrupting intermolecular networks.

Chewiness, representing the energy expenditure during mastication to achieve swallowable consistency (Figure 6b), followed similar trends to hardness but with amplified inter-sample differences. The superior chewiness of RG2:1 (222.94 mJ) versus RG1:1 (113.98 mJ) suggested synergistic effects between hardness and viscoelastic recovery properties. This correlation aligned with food rheology principles where chewiness (W) can be mathematically expressed as W = Hardness × Cohesiveness × Springiness. The reduced chewiness in RG1:1 may indicate incomplete starch gelatinization or protein denaturation during processing, resulting in weaker energy dissipation mechanisms during mastication. RG1:2 and RG2:1 was highly chewable, which may have a slight effect on swallowing in dysphagic populations, but their printing properties were improved [20].

Springiness, quantified as the height recovery ratio post-compression (Figure 6c), peaked in RG1:2, implying superior elastic recovery capacity. This phenomenon could be attributed to the formation of a stable 3D network through polysaccharide–protein coacervation, as demonstrated in alginate–caseinate systems [64]. The lower springiness in other formulations may reflect structural defects from excessive thermal processing or incompatible biopolymer phase separation.

Cohesiveness reflects the adhesion and compactness of the sample’s microstructure, indicating the strength of internal bonds [65] (Figure 6d). RG1:2 exhibited the highest cohesiveness, suggesting the best deformation recovery and resistance to external force-induced deformation. This property reduced the likelihood of food disintegration during swallowing, which was important for dietary safety in patients with swallowing disorders. Thus, diets for individuals with swallowing difficulties should maintain a sufficiently high cohesiveness to ensure food safety [66].

In general, the enhanced textural profiles, particularly in RG2:1 and RG1:2, demonstrated critical structure–function relationships for food manufacturing applications. Improved network integrity directly correlated with shape retention capabilities in emerging technologies like 3D food printing [67]. These findings emphasized the necessity of formulation-specific processing optimization to balance textural parameters with functional requirements.

### 3.7. Sensory Evaluation of 3D-Printed Gel Samples

**Figure 7:**
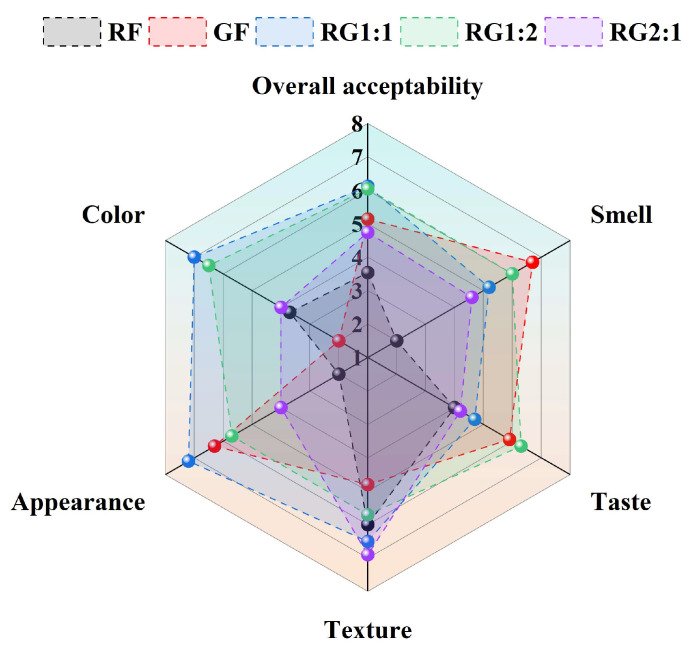
Sensory evaluation results of 3D-printed gel samples.

The results of the sensory evaluation were presented in Figure 7. As illustrated in Figure 7, both the pale yellowish-white color of the GF group (sensory rating 2.0 points) and the excessively dark reddish-brown color of the RF group (sensory rating 3.7 points) were found to have a negative impact on visual perception. The compound system significantly improved the apparent color of the gel, consistent with the findings in Table 4. The odor of GF (6.7 points) and RG1:2 (6.0 points) were more readily accepted, which could be attributed to the mild fragrance of the GBRF, helping to mitigate the bean-like odor emitted by the RABF. Surprisingly, testers showed a stronger preference for the taste of RG1:2 (6.5 points), possibly due to the delicate mouthfeel of GBRF, which contrasted with the deeper, more intense flavor profile of RABF. When blended in appropriate proportions, RG1:2 provided a more balanced and appealing taste. Regarding texture and appearance, RG1:1 received higher ratings of 6.5 and 7.2 points, respectively, which may be due to their higher apparent viscosity, ensuring that the lower layers of the material can adequately support the upper layers and maintain the stability of the printed structure, thus enhancing its aesthetic appeal [62]. This was further corroborated by the rheological measurements. The overall conclusion, based on the composite scores, suggested that both RG1:1 (6.12 points) and RG1:2 (6.04 points) were more readily acceptable, further indicating that the combination of GBRF and RABF may offer promising potential for future 3DFP product development of cereal–legume starch-based gels.

## 4. Conclusions

This study explored the 3D printing performance of cereal–legume starch-based gels formulated with RABF and GBRF, highlighting the potential of these materials for personalized and functional food production. The rheological analysis revealed that the gels exhibited pseudoplastic behavior, with the RG1:2 formulation showing optimal color properties and the RG2:1 formulation demonstrating superior printing fidelity and structural stability. The microstructure analysis indicated that the interaction between RABF and GBRF components significantly influenced the gel network, with higher starch content enhancing the gel’s mechanical strength. Texture analysis further confirmed that the RG2:1 formulation exhibited the highest hardness, while RG1:2 showed the best springiness, suggesting that the ratio of RABF to GBRF can be tailored to achieve desired textural properties. According to the sensory evaluation, RG1:1 and RG1:2 exhibit better sensory qualities. Overall, the findings demonstrated that the combination of RABF and GBRF in specific ratios can enhance the printability and textural properties of cereal–legume starch-based gels, making them suitable for 3D food printing applications. This study provided a foundation for further research into the development of personalized and functional foods using 3DFP technology, with potential applications in catering to specific dietary needs and preferences. Moreover, the ideal application scenarios for all the blended formulations will be also investigated in future studies.

## Figures and Tables

**Table 1:** Experimental formulations of the gel for 3D printing.

Formulations	Ingredients of Base Material	Mass of Base Material (g)	Mass of XG-GG (g)	Mass of Distilled Water (g)
RF	RABF	36	3	108
GF	GBRF	36		
RG1:1	RABF/GBRF (1:1)	18:18		
RG1:2	RABF/GBRF (1:2)	12:24		
RG2:1	RABF/GBRF (2:1)	24:12		

Note: RABF, red adzuki bean flour; GBRF, germinated brown rice flour; All formulations are standardized at 73.47% water and 26.53% solids (RABF and/or GBRF and XG-GG); colloid refers to a mixture of xanthan gum and guar gum.

**Table 2:** Sensory scoring criteria.

Sensory Item	Scoring Criteria	Score
Color	Too light or too dark, failure to stimulate appetite	0–2
	Normal color and even distribution	3–5
	Evenly distributed and can arouse appetite	6–8
Smell	Unacceptable odor	0–2
	No odor or light aroma	3–5
	Strong aroma	6–8
Taste	Unacceptable taste or no taste	0–2
	Light sweet taste, within the acceptable range	3–5
	Sweet and delicious taste	6–8
Texture	Print lines are rough or broken in large areas	0–2
	Print lines are even or have some small breaks	3–5
	Printed lines are even and smooth, with no obvious fracture on the surface	6–8
Appearance	Large areas of missing parts on the surface and accumulation at the corners	0–2
	Relatively slight depressions, a small amount of corner accumulation, and a clear shape	3–5
	No obvious dent on the whole, no corner accumulation, and with a distinct honeycomb structure	6–8
Overall acceptability	Overall evaluation is poor	0–2
	Overall evaluation is average	3–5
	Overall evaluation is good	6–8

**Table 3:** The fitted parameters of *n* and *K* shown in Equation (1).

Group	Non-Newtonian Index (*n*)	Consistency Factor (*K*)	*R* ^2^
RF	0.281 ± 0.01 ^c^	538.967 ± 14.97 ^b^	0.984
GF	0.246 ± 0.01 ^b^	899.287 ± 87.83 ^d^	0.997
RG1:1	0.209 ± 0.01 ^a^	994.867 ± 96.74 ^e^	0.996
RG1:2	0.213 ± 0.01 ^a^	500.397 ± 61.67 ^a^	0.991
RG2:1	0.256 ± 0.01 ^b^	704.853 ± 92.73 ^c^	0.999

Note: Different letters in the same column indicate significant differences between samples (*p* < 0.05); the meanings of the symbols for gel types are detailed in Table 1.

**Table 4:** Color parameters (L*, a*, and b*) of gels and actual color of 3D-printed samples.

Group	L*	a*	b*	ΔE*	Actual Color
RF	54.98 ± 0.80 ^e^	5.77 ± 0.12 ^a^	8.88 ± 0.14 ^d^	0.677 ± 0.60 ^b^	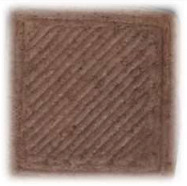
GF	76.17 ± 0.52 ^a^	1.67 ± 0.18 ^d^	12.47 ± 0.36 ^a^	0.623 ± 0.66 ^c^	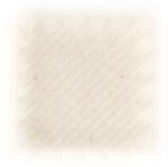
RG1:1	59.43 ± 1.60 ^c^	4.73 ± 0.14 ^b^	9.30 ± 0.22 ^c^	0.403 ± 0.22 ^d^	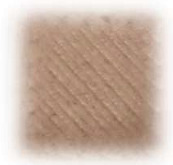
RG1:2	62.36 ± 0.50 ^b^	4.28 ± 0.24 ^c^	9.60 ± 0.32 ^b^	0.603 ± 0.86 ^c^	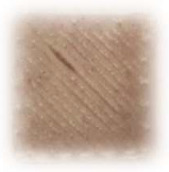
RG2:1	57.70 ± 1.02 ^d^	5.72 ± 0.35 ^a^	9.65 ± 0.45 ^b^	1.872 ± 1.39 ^a^	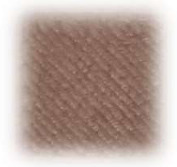

Note: Different letters in the same column indicate significant differences between samples (*p* < 0.05). The meanings of the symbols for gel types are detailed in Table 1.

**Table 5:** SSI values for evaluating the printing fidelity of 3D-printed gel.

Groups	SSI (%)				
	0.5 h	1 h	1.5 h	2 h	Mean ± SD
RF	97.56 ± 0.31 ^c^	96.98 ± 0.12 ^d^	94.79 ± 0.04 ^d^	97.17 ± 0.22 ^d^	96.63 ± 1.14 ^b^
GF	99.21 ± 0.11 ^b^	99.87 ± 0.06 ^a^	99.69 ± 0.16 ^a^	99.92 ± 0.08 ^a^	99.68 ± 0.31 ^a^
RG1:1	99.20 ± 0.07 ^b^	98.44 ± 0.05 ^c^	99.14 ± 0.11 ^b^	98.88 ± 0.10 ^c^	98.92 ± 0.32 ^a^
RG1:2	99.83 ± 0.05 ^a^	98.80 ± 0.05 ^b^	97.55 ± 0.10 ^c^	99.79 ± 0.05 ^a^	98.99 ± 0.97 ^a^
RG2:1	99.55 ± 0.09 ^ab^	98.75 ± 0.04 ^b^	99.67 ± 0.09 ^a^	99.51 ± 0.07 ^b^	99.37 ± 0.39 ^a^

Note: Different letters in the same column indicate significant differences between samples (*p* < 0.05). The meanings of the symbols for gel types are detailed in Table 1.

## Data Availability

The original contributions presented in the study are included in the article; further inquiries can be directed to the corresponding authors.
